# *In vitro* and *in silico* studies on clinically important enzymes inhibitory activities of flavonoids isolated from *Euphorbia pulcherrima*

**DOI:** 10.1080/07853890.2022.2033826

**Published:** 2022-02-03

**Authors:** Abdur Rauf, Muslim Raza, Muhammad Humayun Khan, Hassan A. Hemeg, Yahya S. Al-Awthan, Omar Bahattab, Sami Bawazeer, Saima Naz, Faika Basoglu, Muhammad Saleem, Majid Khan, Hosseini Seyyedamirhossein, Mohammad S. Mubarak, Ilkay Erdogan Orhan

**Affiliations:** a Department of Chemistry, University of Swabi, Khyber Pakhtunkhwa, Pakistan; b Department of Chemistry, Bacha Khan University Charsadda, Khyber Pakhtunkhwa, Pakistan; c Institute of Chemical Sciences, University of Peshawar, Khyber Pakhtunkhwa, Pakistan; d Department of Medical Laboratory Technology, College of Applied Medical Sciences, Taibah University, Medina, Saudi Arabia; e Department of Biology, Faculty of Sciences, University of Tabuk, Tabuk, Saudia Arabia; f Department of Biology, Faculty of Science, Ibb University, Ibb, Yemen; g Department of Pharmacognosy, Faculty of Pharmacy, Umm Al-Qura University, Makkah, Saudi Arabia; h Department Biotechnology and Microbiology, Bacha Khan University Charsadda, Khyber Pakhtunkhwa, Pakistan; i European University of Lefke, Faculty of Pharmacy, Department of Pharmaceutical Chemistry, Lefke, Northern Cyprus, Mersin, Turkey; j Department of Chemistry, Ghazi University, Punjab, Pakistan; k H.E.J. Research Institute of Chemistry, University of Karachi, Karachi, Pakistan; l Department of Chemistry, Indiana University, Bloomington, IN, USA; m Department of Chemistry, The University of Jordan, Amman, Jordan; n Department of Pharmacognosy, Faculty of Pharmacy, Gazi University, Ankara, Turkey

**Keywords:** *Euphorbia pulcherrima*, urease, tyrosinase, antiglycation, phosphodiesterase, docking analysis

## Abstract

**Introduction:** The genus *Euphorbia* is known to contain diterpenoids, and several isolated compounds which exhibited biological activities including significant multidrug resistance reversal effects. This work is focused on the isolation, *in vitro* and *in silico* studies of two natural bio-active flavonoids (**1** & **2**) isolated from *Euphorbia pulcherrima* bark for the very first time.

**Methods:** The phytochemical investigation resulted in the identification of two flavonoids: 3,5,7-trihydroxy-2-(4-hydroxy-3-methoxyphenyl)-6-methoxy-4H-chromen-4-one **(1)** and 2-(3,4-dihydroxyphenyl)-3,5,7-trihydroxy-6-methoxy-4H-chromen-4-one **(2)**, which were isolated for the first time from *Euphorbia pulcherrima*.

**Results:** The chemical structures of the two isolated compounds were confirmed by ^1^H NMR, ^13^C NMR, and ESI-HRMS spectral data. The Bioactivity activity of these compounds was evaluated; results revealed that compounds 1 & 2 exhibit promising urease inhibitory potential with IC50 values of 15.3 ± 2.13 μM and 19.0 ± 2.43 μM, respectively, whereas the positive control thiourea had an IC_50_ of 21.0 ± 0.23 μM. Similarly, these compounds were also evaluated against the tyrosinase enzyme; results showed that compound **1** displays significant inhibitory activity with an IC_50_ value of 48.7 ± 2.19 μM, whereas compound 2 exhibited a moderate effect with an IC50 value of 74.8 ± 1.79 μM, when compared with the standard (alpha-kojic acid, IC_50_ = 47.6 ± 0.67 μM). Additionally, compounds **1** and **2** also exhibited anti-glycation and phosphodiesterase inhibitory activities.

**Conclusion:** Studies dealing with the drug like properties such as *in silico* screening (docking study) was also carried out to discover the structural features of both compounds **1** and **2**. Results indicated that the docking scores of compounds **1** and **2** are in agreement with their IC_50_ values.

**Key messages**
- Isolation and characterization of two bioactive flavonoids (**1** and **2**) from *Euphorbia pulcherrima*.
- *In silico* and *in vitro* enzyme inhibition studies were conducted to identify the therapeutic potential of flavonoids **1** and **2**.
- Drug-like properties were calculated to discover important pharmacophoric features.

## 1. Introduction

*Euphorbiaceae*, also called ‘Euphorbias or spurge family,’ is a large flowering family among the Anthophyta with more than 300 genera and 5000 species that spread worldwide. Members of this family are rich in secondary metabolites. Genus *Euphorbia* is the largest of this family, and regarded as a kingdom of medicinal plants in few countries of Asia such as Pakistan and India. In addition, the genus *Euphorbia* with various species is used in traditional medicine to cure different ailments. Some species in this family are toxic, while others can be used for things such as poinsettia (*E. pulcherrima*), spurges, crowns of the throne, and milk bush. However, all of these species contain latex as an abundant source of secondary metabolites [1]. Furthermore, the genus *Euphorbia* is known to contain diterpenoids, and several isolated compounds exhibited biological activities including significant multidrug resistance reversal effects [2]. For instance, the essential oil from *E. hirta* L., which is also called ‘asthma plant’ has been traditionally used to cure asthma. This oil consists of various components such as triterpenes, phytosterols, tannins, polyphenols, and flavonoids, which can be used for different ailments. Besides, the essential oil has been employed as mosquito repellent, thus preventing malaria [3].

On the other hand, *E. kansui* L.’s dried roots from Euphorbiaceae have been used as a herbal remedy for edoema, ascites, and cancer in Chinese traditional medicine [3]. *Euphorbia hirata* L., locally known as ‘Dhudi,’ is an annual hairy plant, mostly found in waste places and open grasslands, contains alkaloids, saponins, flavonoids, and tannins, and is used to cure of different diseases including gastrointestinal disorders, kidney stones, bronchial ailments, diabetes, and respiratory diseases [4]. Similarly, *E. pulcherrima*, called ‘poinsettia, Christmas star or Christmas flower,’ grows in the Pacific coast of America and is distributed in southern Mexico as well as Guatemala. It is also found throughout Nepal, mainly in the mountains area, and in various countries of Asia [5]. The aerial parts of *E. pulcherrima* have been used in folk medicine to treat various ailments such as skin diseases, normal and slow-transit constipation, and increase milk secretion in nursing mothers. On the other hand, some other genus Euphorbia species exhibit useful activities such as central analgesic properties, anti-inflammatory, antipyretic, strong sedative effect, and anti-depressant [6]. Furthermore, numerous pharmacological studies indicated that family euphorbiaceae can be used to cure central nervous system, as analgesic, antipyretic, anti-inflammatory, anti-covulsant, hypnotic, and neuroleptic among other things [7].

On the basis of the preceding discussion, and in light of the wide pharmacological profile of the family *Euphorbiaceae*, the aim of current work was to isolate phytochemicals which can exhibit biological activities. In spite of the extensive phytochemical research that has been conducted on family *Euphorbiace*, thorough investigation on the phytochemical constituents is still required to increase the knowledge related to this family. Consequently, the present study resulted in the isolation of 3,5,7-trihydroxy-2-(4-hydroxy-3-methoxyphenyl)-6-methoxy-4*H*-chromen-4-one (**1**) and 2-(3,4-dihydroxyphenyl)-3,5,7-trihydroxy-6-methoxy-4*H*-chromen-4-one (**2**) for the first time from the bark *Euphorbia pulcherrima*. The pure and fully characterized compounds **1** and **2** were subjected to different biological activities such as urease, tyrosinase, phosphodiesterase, and anti-glycation inhibition studies.

## 2. Materials and methods

### 2.1. Plant material

Fresh barks from *Euphorbia pulcherrima* were collected in the month of August 2018 from the University of Peshawar (Khyber Pakhtunkhwa, Pakistan) and were cleaned, washed, and crushed.

Dr Barkatullah, a botanist at the Department of Botany, University of Peshawar, Pakistan, identified and authenticated the plant. A voucher specimen (PUP545) was deposited at the herbarium located at the Department of Botany, University of Peshawar, Pakistan.

### 2.2. Reagents and equipment

Chemicals and reagents used throughout this work were obtained from commercial sources and used as received. Urease (from jack beans) solution in 50% glycerol, 1000 U/mL for biochemistry (EC 3.5.1.5), phosphodiesterase I from *Crotalus adamanteus* venom (P3134), tyrosinase from mushroom (T3824), urea (U4883), thiourea, urea (U4883), and Tris-HCl (T5941) were purchased from Sigma-Aldrich (St. Lewis, MO, USA). ELISA plates analyzer were SpectraMax M2, purchased from Molecular Devices (Sunnyvale, CA, USA). We recorded the NMR spectra on a 500 MHz Varian Inova spectrometer (Palo Alto, CA, USA), with tetramethylsilane (TMS) as an internal standard. Chemical shifts are expressed in *δ* units, and 1H − 1H coupling constants (*J* values) are given in hertz. High resolution mass spectra (HRMS) were acquired by electrospray ionization (ESI) technique using a Hewlett − Packard 5890 Series II gas chromatograph (Thermo Electron Corporation) equipped with a DB-5 capillary column and coupled to a MAT-95X magnetic-sector mass spectrometer (Poway, CA).

### 2.3. Extraction and isolation

*Euphorbia pulcherrima* bark was dried in the shade for 20 days. Approximately 8.12 kg of dried barks was turned into powder with a grinder. The powdered bark material was subjected to cold extraction using a polar solvent (methanol, 100 L). The dilute methanol extract was concentrated and dried under reduced pressure by means of rotary evaporation to afford 170.54 g. The crude extract was further subjected to extractions with solvents of different polarity including *n*-hexane (10 L), chloroform (12 L), ethyl acetate (8 L), and 1-butanol (8 L) to give different fractions. On the basis TLC profile, the chloroform fraction (34.2 g) was selected for further processing because it contains maximum number of compounds. Approximately 20 g of the chloroform fraction was loaded onto a silica gel column eluted with a solvent system of *n*-hexane and ethyl acetate (100:0**−**0:100). On the basis of the TLC profile, 80 sub-fractions (Fr-1 to Fr-80) were obtained. Defatting was achieved by elution with *n*-hexane (100 mL). Then the column was eluted with mixtures of *n*-hexane: ethyl acetate (100:0**–**30:80) to afford the sub-fraction of a brown colour. This sub-fraction was further run through preparative chromatography with the solvent system *n*-hexane and ethyl acetate (60:40), which led to the isolation of compounds **1** (0.47 g) and compound **2** (0.51 g). Different spectroscopic techniques were employed to elucidate the chemical structure of these compounds (**1** and **2**). Their spectral data were similar to those previously reported in the literature [8].

### 2.4. Characterization of compounds 1 and 2

Structures of the isolated compounds **1** and **2** were confirmed by mass spectrometry (MS) and NMR spectral data. They were identified by comparison of their spectroscopic properties with literature data [8]. Thus, the mass spectra displayed the correct molecular ion peaks for which the measured high resolution (HRMS) data are in good agreement with the calculated values. In addition, structures were verified by comparing their NMR data with those reported in the literature. These known compounds were isolated for the first time from the bark extract of this species.

#### 2.4.1. Spinacetin (1)

Spinacetin, compound **1**, was isolated as an amorphous brownish solid. High-resolution ESI − MS (HRMS) exact mass *m*/*z*: calculated for C_17_H_14_O_8_ [M]^+^ 346.0689, found 346. 0683. ^1^H-NMR (500 MHz, DMSO-d_6_): *δ* 12.26 (2H, s, OH), 9.94 (2H, s, OH), 8.07 (1H, d, *J*_5’, 6′_ = 8.0 Hz, H-6), 7.78 (1H, dd, *J*_5’,6′_ = 8.0 Hz, 7.8 Hz, H-5′), 7.74 (1H, d, *J*_2’,5′_ = 4.0 Hz, H-2′), 7.30 (s, 1H, H-8), 2.76 (3H, s, –OCH_3_-6), 2.03 (3H, s, –OCH_3_-4′).

#### 2.4.2. Patuletin (2)

Patuletin, compound **2**, was isolated as an amorphous yellow powder. High-resolution ESI − MS (HRMS) exact mass *m*/*z*: calculated for C_16_H_12_O_8_ [M]^+^ 332.2617, found 332.2610. ^1^H-NMR (500 MHz, DMSO-d_6_): *δ* 12.27 (2H, s, OH), 9.95 (2H, s, OH), 8.08 (1H, d, *J*_5’, 6′_ = 8.0 Hz, H-6), 7.79 (1H, dd, *J*_5’,6′_ = 8.0 Hz, 7.91 Hz, H-5′), 7.75 (1H, d, *J*_2’,5′_ = 4.0 Hz, H-2′), 7.32 (s, 1H, H-8), 2.76 (3H, s, –OCH_3_), 12.39 (H, s, –OH). Th**e** spectral data of both compounds (**1** and **2**) were similar to those previously reported in the literature [8].

### 2.5. Urease inhibition activity of compounds 1 and 2

*In vitro* urease inhibitory activity of purified compounds **1** and **2** was investigated spectrophotometrically in a 96-well plate according to published procedures [9]. In this assay, 25 μL of Jack bean (*Canavalia ensiformis*) urease (1 U/well) and 5 µL solution of studied compound (0.5 mM) in Tris-HCl buffer (pH = 6.8) were incubated at 30 °C for 15 min in 96-well plate. The mixture was re-incubated for 15 min at 30 °C with urea 55 µL (100 mM). Afterwards, 45 µL of phenol (1% *w/v* phenol and 0.005% *w/v* sodium nitroprusside) and 70 µL of alkali reagents (0.5% *w/v* sodium hydroxide and 0.1% sodium hypochlorite) were added, and the plates were re-incubated for another 50 min. The urease assay was monitored on regular basis with hydrolysis of urea and production of ammonia; thiourea was employed as the standard inhibitor of urease The fast change in the absorbance (OD, optical density) was checked at 630 nm using an ELISA plate reader (Spectra Max M2, Molecular Devices, CA, USA). The percentage inhibition was calculated according to the formula given below:

% Inhibition=100−(OD test /OD control)×100

### 2.6. Phosphodiesterase 1 inhibition assay

A mixture of 15 μL of phosphodiesterase-1 (E.C 3.1.4.1), 20 μL of magnesium acetate (30 mM), 97 μL Tris-HCl buffer (pH = 8.8), 97 μL Tris-HCl buffer (pH = 8.8), and 8 μL of test compound was placed in 96-well plates to make a final concentration of 0.000742 U/well; the mixture was incubated again in an ELISA for 30 min at 37 °C, followed by the addition of 0.33 mM bis (*p*-nitrophenyl) phosphate. The enzymes activity was measured spectrophotometrically at 410 nm using a microplate reader (SpectraMax, Molecular Devices, Sunnyvale, CA, USA), as reported in the literature [10].

### 2.7. Tyrosinase inhibition

The evaluation of the tyrosinase inhibitory activity of isolated compounds **1** and **2** was performed spectrophotometerically using microplate in a 96-well, according to a published method [11]. According to this method, the tested compounds (10 µL) was carefully transfer into a 96-well microplate, mixed with 10 µL mushroom tyrosinase and buffer solution 60 µL. After incubation, 20 µL of 3,4-dihydroxy-l-phenylalanine (L-DOPA) in phosphate buffer was blended, and some amount of dopachrome was added into the reaction mixture. The effect was checked by spectrophotometry at 450 nm with (OD480) (optical density, OD). EZ-Fit software was used to measure the IC_50_ values in µM. The % inhibition was calculated by using following formula.

$$\text{Percent inhibition}=100-\frac{\text{OD test well}}{\text{OD control}}\times 100$$

### 2.8. Anti-glycation inhibition

Assessment of the anti-glycation inhibition of compounds **1** and **2** was undertaken according to published procedures with slight changes [12,13]. Briefly, triplicate mixtures of 14 mM magnesium oxide (MGO), bovine serum albumin solution (BSA) (10 mg/mL) and 0.1 M phosphate buffer (pH 7.4) along with NaN_3_ (30 mM) were incubated in such a way that each well of 96-well plate has 50 µL MGO, 50 µL BSA solution at 37 °C for 9 days in the absence and presence of various amounts of test sample (compounds). After incubation, glycation of protein was measured using fluorescence (emission, 440 nm; excitation, 330 nm), against a blank, and using a microtiter plate spectrophotometer (Spectra Max, Molecular Devices, Sunnyvale, CA). Rutin was used as positive control (IC_50_ = 294 ± 1.50 µM ± SEM).

### 2.9. Molecular docking studies

Tyrosinase, PDB ID: 2Y9W, protein structures (urease, PDB ID: 4GY7, PDB ID:3VO3) and bovine serum albumin were downloaded from (PDB) protein data bank. The targeted sequence for the other enzymes, phosphodiesterase-I (PDE-1 from snake venom) was retrieved from the UniPort database [14], with the accession number of J3SEZ3. BLAST against PBD found the optimal template [15]. The optimal template was selected based on sequence similar to the PDE-1 enzyme structure from Mus musculus (PDB ID: 4GTW). With the help of Bio edit software and MODELLER 9.12, target template sequence alignment and 3-D model creation were completed. [16]. Swiss PDB viewer v4.1.0 software was employed to refine the projected models’ energy [17]. Similarly, ProSA 27 [18] and Procheck online servers [19] were used to assess the models, and the best predicted model was chosen for future docking investigations. The Swisspdb viewer v4.1.0 software was used to optimize the crystal structure’s geometry [20]. Chem sketch [21] and Avogadro’s program [22] were used to construct compounds **1** and **2** as well as standard structures for docking. We employed Autodockvina [23] to conduct docking experiments, and technique improvements of the docking software were done first, and Autodockvina [24] was linked with PyRex tools. Removal of solvent molecules, hydrogen addition, and computation of gasteiger charges were all completed [25]. Autodockvina was used to dock with all of the default settings [26,27]. LIGPLOT + version v.1.4.5 [28] and PyMOL version 1.7.2 [29] were employed to perform interaction studies on docked complexes.

### 2.10. ADMET properties calculation

Med Chem Designer v.3 was employed to evaluate the physicochemical characteristics of compounds **1** and **2**. Drug permeability (S + log*P*), distribution (S + log*D*), topological polar surface area (TPSA), MLog*P*, molecular weight (M. wt), and the total amount of nitrogen and oxygen atoms were all assessed according to literature methods [30]. These metrics can disclose details about a compound's absorption, distribution, metabolism, excretion, and toxicity (ADMET). These characteristics of molecules are critical for determining the therapeutic effectiveness and appropriateness [31].

## 3. Results and discussion

The family *Euphorbiaceae* has long been used in traditional medicine. In this respect, the essential oil from *E. hirta* is used to cure asthma, because it consists of various components including triterpenes, phytosterols, tannins, polyphenols, and flavonoids, which can be used to treat different ailments. The essential oil has also been used as mosquito repellent, thus preventing malaria [3]. Similarly, *E .kansui* L.'s dried roots from Euphorbiaceae have been used as a herbal remedy for edoema, ascites, and cancer in Chinese traditional medicine [3]. Moreover, *E. hirata* L., is used for the cure of different diseases including gastrointestinal disorders, kidney stones, bronchial ailments, diabetes, and respiratory diseases [4].

Natural products have received more attention in recent years as researchers look for new medications using cutting-edge technologies such as high-throughput screening [32]. Compounds **1** and **2** were tested for urease, tyrosinase, and phosphodiesterase inhibition, among other enzymes. When compared to the standard thiourea (IC_50_ = 21.0–0.21 µM), both compounds exhibited significant inhibitory action against urease with IC_50_ = 15.3–2.13 and 19.0–2.43 µM, respectively.

This action might be attributed to the presence of two hydroxyl groups (–OH) on ring 3 of flavonoids, which could interact with residues near the nickel atoms in the urease active site. In summary, these two hydroxyl groups may be the active pharmacophore in both molecules. Similarly, when compared to the standard kojic acid (47.6, 0.67 M), both compounds showed substantial to moderate inhibition against tyrosinase, with IC_50_ values of 48.7 and 74.8 µM, respectively. On the other hand, the standard drug showed excellent effect with IC_50_ =47.6 ± 0.67 µM, compound **2** demonstrated strong action with an IC_50_ value of 95.2 at 4.14 µM, whereas compound **1** exhibited substantial activity with an IC_50_ value of 148.7 at 1.09 µM. Both compounds were also evaluated for anti-glycation activity; results revealed that compound **2** exhibits strong anti-glycation activity with an IC_50_ value of 244.8 at 1.79 µM, whereas compound **1** was found to be inactive when compared to the standard, rutin (295, 3.14 µM). Listed in Table 1 are results of enzyme inhibitory activities of compounds **1** and **2**.

**Table 1.** Enzyme inhibitory screening of compounds **1** and **2** isolated from *E. pulcherrima*.

Code	IC_50_ ± S.E.M. (µM) (urease)	IC_50_ ± S.E. (µM) (tyrosinase)	IC_50_ ± S.E.M. (phosphodiesterase)	IC_50_ ± S.E. (µM) (antiglycation)
H16	15.3 ± 2.13	48.7 ± 2.19	148.7 ± 1.09	NA
H5	19.0 ± 2.43	74.8 ± 1.79	95.2 ± 4.14	244.8 ± 1.79
STD	21.0 ± 0.21 (thiourea)	47.6 ± 0.67 (kojic acid)	265 ± 2.24 (EDTA)	295 ± 3.14 (rutin)

S.E.M.: standard error of the mean; IC_50_: minimum inhibitory concentration; STD: standard.

**Figure 1.**
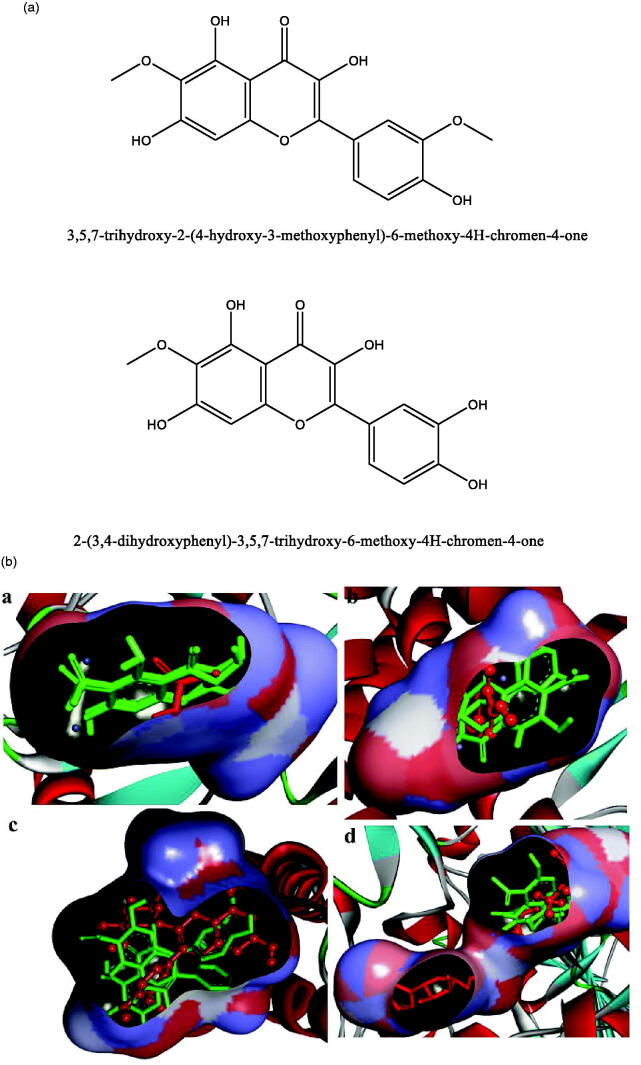
(a) Chemical structures of isolated compounds from *E. pulcherrima*. (b) Illustration of predicted docked poses of compounds **1** and **2** (indicated by green colour sticks) in the binding pocket of urease (a), tyrosinase (b), bovine serum albumin (c), and phosphodiesterase-I (d). All predicted conformations were created at the binding site of crystal structures, whereas existing co-crystallized compounds are bonded in the active site.

Listed in Table 1 are results of the *in vitro* enzymatic activities of compounds **1** and **2**. Results reveal that these two compounds exhibit significant inhibitory activities due to their distinct structural features. In addition, docking studies were conducted to evaluate the binding pattern of compounds **1** and **2** at the active sites of urease, tyrosinase, bovine serum albumin, and phosphodiesterase-I. Tables 2 and 3 provide docking results for compounds **1** and **2** as well as the reference compounds. Based on hydrogen and hydrophobic interactions, the best-docked shape of compound **1** was investigated. Results showed that the docking energies of compound **1** are superior to those of the reference (thiourea) and other compounds. This implies that certain aspects in the structure of compound **1** are responsible for affecting the biological activity. Docked conformations of compounds **1** and **2** and thiourea are shown in Figure 1(a,b).

**Figure 2.**
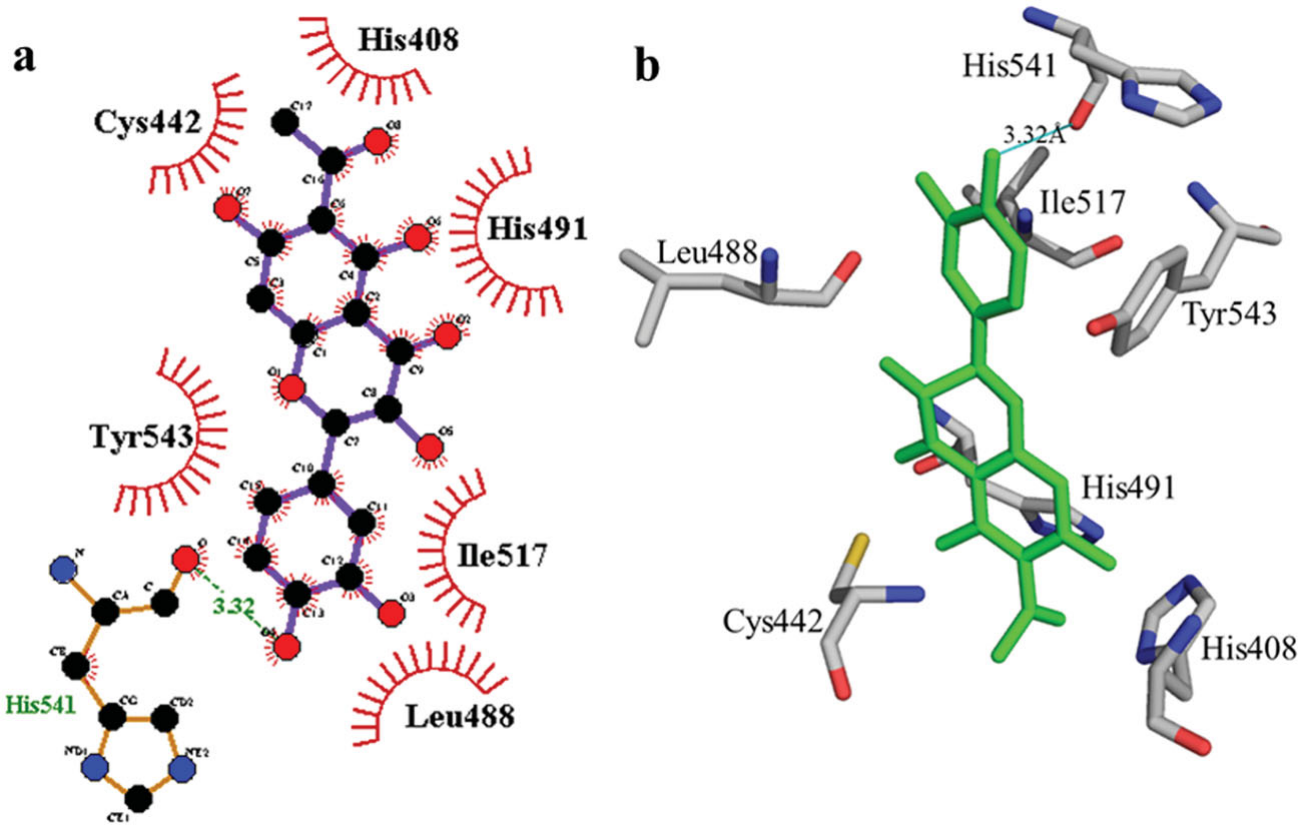
Interactions of compound **1** with the binding pocket of urease enzyme in 2D (a) and 3D (b). Hydrophobic interactions are depicted as half-moons, whereas dotted green lines with distances in angstrom represent hydrogen bonding.

**Table 2.** Molecular docking details of isolated compounds **1** and **2** and against urease and tyrosinase enzymes.

Compound		Hydrogen bonding residues and distance		Hydrophobic interacting residues	B. affinity (kcal/mol)
**1**		His541	3.32 Å	His408, Cys442, Leu488, His491, Ile517 and Tyr543	–8.7
**2**		Ala439	2.69 Å	Cys405, His408, Thr437, Cys442, Leu488, His491, Ile517, His541 and Tyr543	–8.7
Thiourea (standard)					–3.4
**1**	No H bonding			Gly62, Phe90, Trp93, Val262, Phe292 and His296	–7.6
**2**	No H bonding			Phe90, Val262, Phe292, His295 and His296	–7.5
Kojic acid (standard)					–5.1

**Table 3.** Docking details of BSA and phosphodiesterase-I receptors with compounds **1** and **2**.

Compound		Hydrogen bonding residues and distance		Hydrophobic interacting residues	B. affinity (kcal/mol)
**1**		His145 Glu424 Ser428	3.06 Å 2.84 Å 2.75 Å	Leu189, Thr190, Ala193, Arg196, Lys431, Tyr451 and Arg458	–8.8
**2**		Arg194 Ser428 Arg458	3.09 Å 2.34 Å 2.99 Å	His145, Val188, Thr190, Ser191, Val425 and Ile455	–8.5
Rutin (standard)					–9.4
**1**	Tyr378 Thr455 Arg456		3.21 Å 2.90 Å 2.83 Å	Lys184, His309, Leu363, GLu379, Gly380, Pro381, Trp446, Met447 and Thr461	–8.4
**2**	Lys184, Trp446 His462		3.34 Å 3.01 Å 3.05 Å 3.16 Å	His309, Leu363, Glu379 and Gly380	–8
EDTA (standard)					–4.9

**Figure 3.**
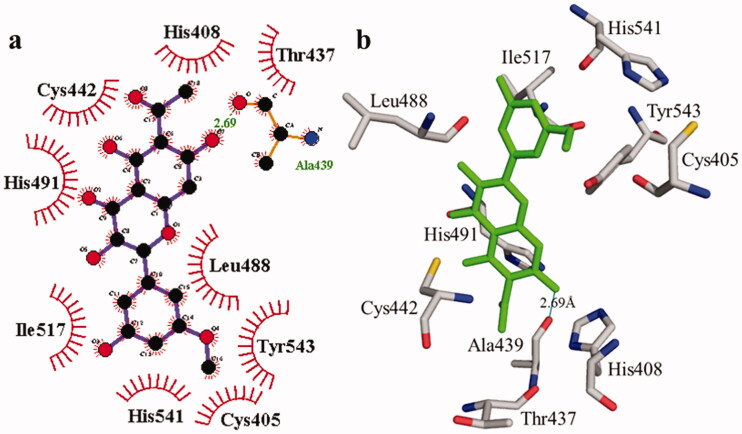
The interactions of the urease enzyme with compound **2** are determined by binding residues. Compound **2** interacts with the active site in 2D (a) and 3D (b).

**Figure 4.**
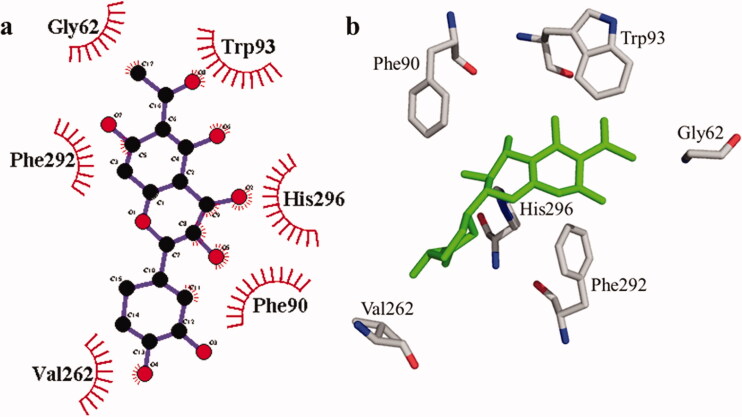
The interactions of the tyrosinase enzyme with compound **1** are determined by binding residues. Detailed interactions of compound **1** with active sites of the enzyme in 2D (a) and 3D (b).

**Figure 5.**
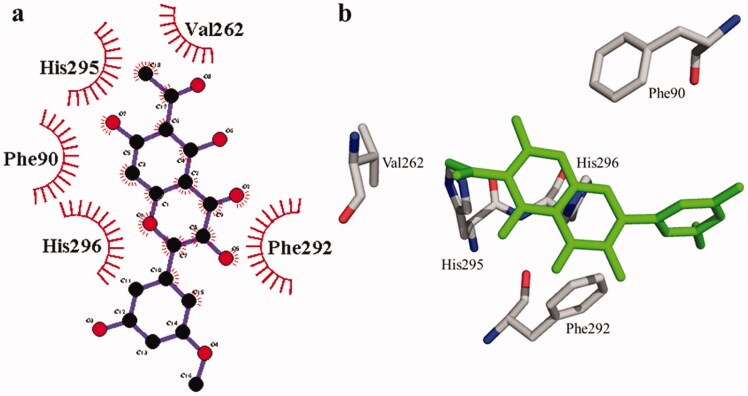
The interactions of the tyrosinase enzyme with compound **2** are determined by binding residues. Detailed interactions of compound **2** with active sites of the enzyme in 2D (a) and 3D (b).

**Figure 6.**
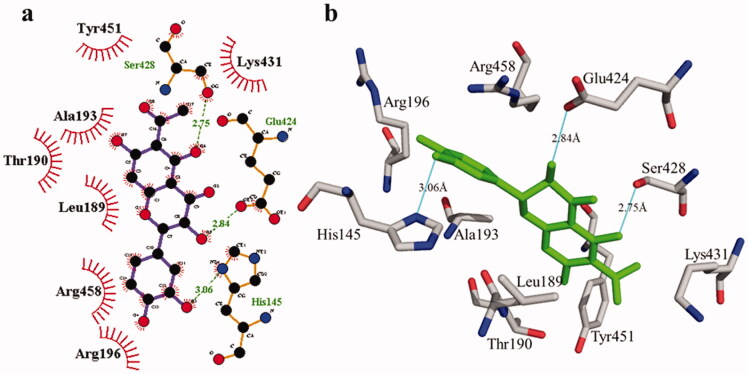
The interactions of bovine serum albumin with compound **1**. Detailed interactions of compound **1** with active sites of the enzyme in 2D (a) and 3D (b).

**Figure 7.**
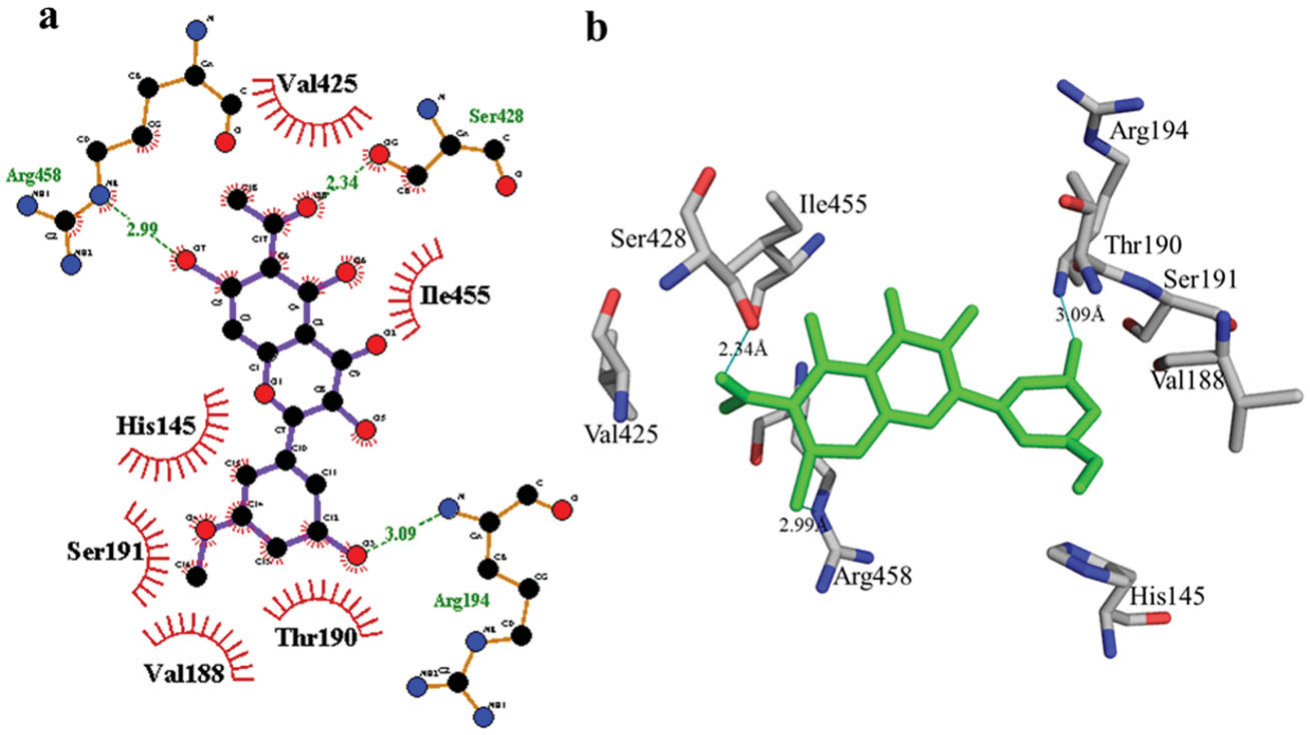
Detail interactions display of compound **2** with bovine serum albumin. Detailed interaction of compound **2** with the active site in 2D (a) and 3D (b).

According to the interaction study, compound **1** forms one hydrogen bond at the binding site of urease enzyme. This hydrogen bond of 3.32 Å length was found in His541 with the –OH group of compound **1** (Figures 1(a,b) and 2). Additionally, the surrounding residues Cys442, His408, Ile517, His491, Leu488, and Tyr543 were examined for the six hydrophobic interactions. Similarly, results showed one hydrogen bonding with Ala439 (2.69 Å), in addition to the hydrophobic interaction with the residues His408, Cys405, Cys442, His491, His541, Tyr543, His541, and Thr437 as displayed in Figure 3. Moreover, the comprehensive interaction of tyrosinase (Figure 4) with compound **1** shows six hydrophobic contacts from nearby residues His296, Phe292, Val262, Trp93, Phe90, Gly62, and Trp93. On the other hand, compound **2** (Figure 5) showed five hydrophobic contacts with His296, Phe90, Phe292, Val262, and His295. Furthermore, the albumin interaction with compound **1** indicates the existence of three hydrogen bonds with the surrounding residues (Ser428, 2.75 Å, Glu424, 2.84 Å, His145, 3.06 Å,), as well as hydrophobic contacts with Thr190, Ala193, Tyr451, Lys431, Arg458, Arg196, and Leu189 as displayed in Figure 6. Furthermore, compound **2** (Figure 7) shows numerous hydrogen bonding (Ser428, 2.34 Å, Arg194, 3.09 Å, Arg458, 2.99 Å), as well as six hydrophobic interactions with Val188, His145, Ile455, Thr190, Val425, and Ser191.

**Figure 8.**
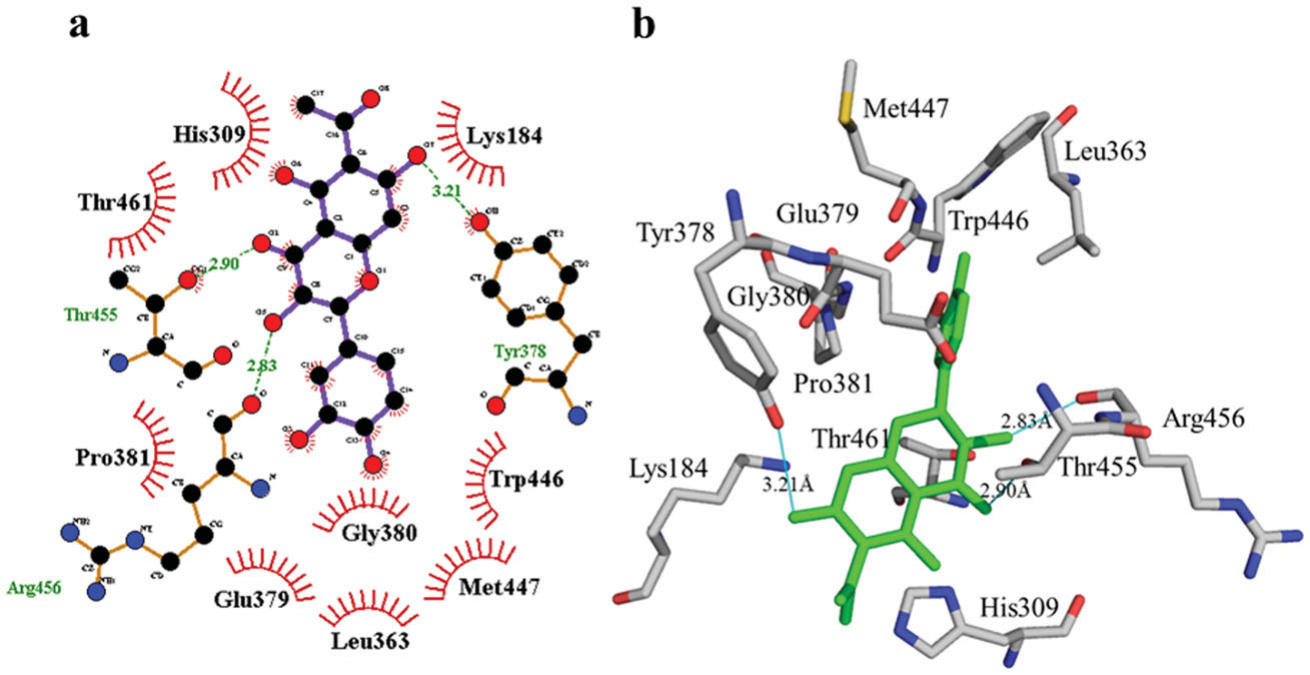
Phosphodiesterase-I interactions with compound **1** are mediated by binding residues. Detailed interaction of compound **1** with the active site is shown in 2D (a) and 3D (b).

**Figure 9.**
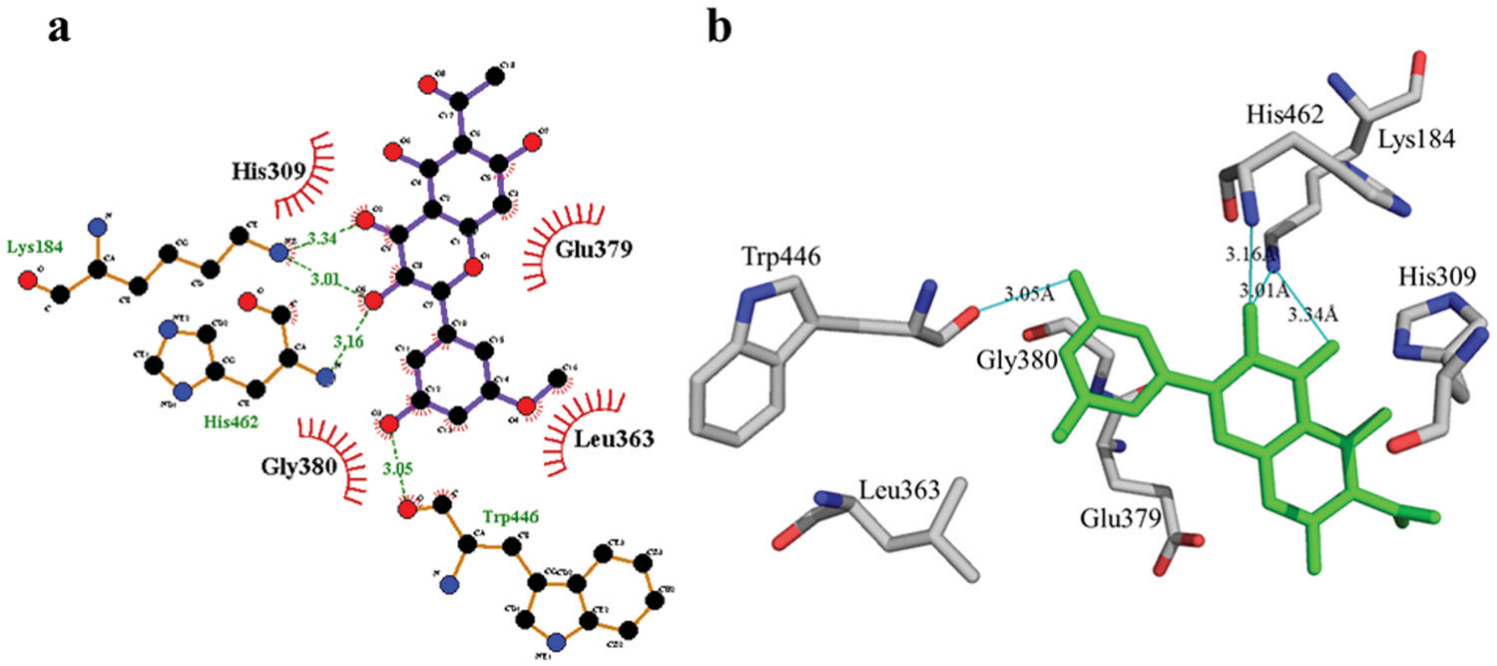
Phosphodiesterase-I interactions with compound **2** are mediated by binding residues. Detailed interaction of compound **2** with the active site in 2D (a) and 3D (b).

The phosphodiesterase-I interaction profile with compound **1** was quite interesting as depicted in Figure 8. Three hydrogen bonding (Thr455, 2.90 Å; Arg456, 2.83 Å, and Tyr378, 3.21 Å) and nine hydrophobic contacts (Leu363, Lys184, GLu379, His309, Gly380, Trp446, Pro381, Thr461, and Met447) were shown. Meanwhile, there are four hydrogen bonds in compound **2**, two of which are formed with Lys184 (3.01 Å and 3.34 Å) whereas the remaining two are formed with the residues His462 (3.16 Å) and Trp446 (3.05 Å). Likewise, four hydrophobic interactions (Figure 9) were also detected with surrounding residues of phosphodiesterase-I (His309, Glu379, Leu363, and Gly380).

Compounds **1** and **2**’s adsorption, distribution, metabolism, excretion, and toxicity (ADMET) characteristics were estimated with the help of Med Chem Designer. Lipophilicity is defined as the logarithm value of the partition coefficient P (logP) between water and octanol (buffer), which describe how the unionized form of compound is partitioned. The total partition of both the ionized and unionized forms of the compound is described by logD. Table 4 shows that compounds **1** and **2** have a log *P* value of less than 5, suggesting that they are hydrophilic. The log*D* values of the compounds differ from those of log*P* when they ionize at different pH levels, and the log*D* value of acidic substances is lower [33]. Moriguchi’s log*P* (Mlog*P*) is a classic measure of a compound’s lipophilicity, which shows how well it penetrates lipid-rich environments from aqueous solutions. MLogP values over 4.15 indicate that the chemical is poorly absorbed [33].

**Table 4.** Prediction of ADMET properties of compounds **1** and **2**.

Compound no.	Mlog*P*	S + log*P*	S + log*D*	Rule of 5	MWt	M_NO	TPSA	HBDH
**1**	–0.415	1.466	–0.652	0	346.295	8	144.52	5
**2**	–0.327	2.118	0.863	0	358.307	8	137.43	4

Compounds **1** and **2** have Mlog*P* values of −0.415 and −0.327, respectively, indicating that they should be easily absorbed. Similarly, these molecules have the potential to make H-bonding with the receptor as determined by the topological polar surface area (TPSA) score. TPSA ratings for compounds **1** and **2** are 144.52 and 137.43, respectively. Formation of additional hydrogen bonds is due to the presence of numerous nitrogen and oxygen atoms. Thus, the present investigation showed that compounds **1** and **2** are hydrophilic due to strong hydrogen bonding and high TPSA score, based on the binding affinity and adsorption, distribution, metabolism, excretion, and toxicity (ADMET) characteristics. Furthermore, analysis of the ADMET characteristics for compounds **1** and **2** shows that they have strong drug-like qualities, which opens the door for further optimization of the compounds under investigation.

In summary, the isolated compounds **1** and **2** demonstrated significant inhibitory potency against urease, tyrosinase, and phosphodiesterase, whereas for antiglycation, compound **1** was inactive. In addition, our findings showed that compounds **1** and **2** exhibit substantial urease inhibitory activity against the reference thiourea. These findings may explain why this plant is used in folk medicine for therapeutic purposes. However, more detailed studies are required to establish the safety and efficacy of these chemicals. Furthermore, research involving animals and toxicological studies on compounds **1** and **2** could also be beneficial.

## Ethical approval

This study was approved by the Ethical Committee of the Department of Pharmacy University Peshawar (UOS) Phrm 432, KPK, Pakistan.

## Data Availability

The data based on the results of the current study are obtained from the corresponding authors upon request.
